# Macrophage and Dendritic Cell Activation and Polarization in Response to *Coccidioides* *posadasii* Infection

**DOI:** 10.3390/jof7080630

**Published:** 2021-08-03

**Authors:** Anh L. Diep, Susana Tejeda-Garibay, Nadia Miranda, Katrina K. Hoyer

**Affiliations:** 1 Quantitative Systems Biology Graduate Programme, University of California Merced, Merced, CA 95343, USA; adiep4@ucmerced.edu (A.L.D.); stejeda-garibay@ucmerced.edu (S.T.-G.); nmiranda8@ucmerced.edu (N.M.); 2 Department of Molecular and Cell Biology, School of Natural Sciences, University of California Merced, Merced, CA 95343, USA; 3 Health Sciences Research Institute, University of California Merced, Merced, CA 95343, USA

**Keywords:** *Coccidioides immitis*, *Coccidioides posadasii*, coccidioidomycosis, Valley fever, innate immunity, macrophage, dendritic cell, polarization

## Abstract

Coccidioidomycosis is a fungal, respiratory disease caused by *Coccidioides immitis* and *Coccidioides posadasii*. The host immune responses that define disease outcome during infection are largely unknown, although T helper responses are required. Adaptive immunity is influenced by innate immunity as antigen-presenting cells activate and educate adaptive responses. Macrophage and dendritic cell (DC) recognition of pathogen surface molecules are critical for *Coccidioides* clearance. We characterize the broad innate immune responses to *Coccidioides* by analyzing macrophage and dendritic cell responses to *Coccidioides* arthroconidia using avirulent, vaccine *Coccidioides* strain NR-166 (*Δcts2/Δard1/Δcts3*), developed from parental virulent strain C735. We developed a novel flow cytometry-based method to analyze macrophage phagocytosis to complement traditional image-scoring methods. Our study found that macrophage polarization is blocked at M0 phase and activation reduced, while DCs polarize into proinflammatory DC1s, but not anti-inflammatory DC2, following interaction with *Coccidioides*. However, DCs exhibit a contact-dependent reduced activation to *Coccidioides* as defined by co-expression of MHC-II and CD86. In vivo, only modest DC1/DC2 recruitment and activation was observed with avirulent *Coccidioides* infection. In conclusion, the vaccine *Coccidioides* strain recruited a mixed DC population in vivo, while in vitro data suggest active innate immune cell inhibition by *Coccidioides*.

## 1. Introduction

*Coccidioides immitis* and *Coccidioides posadasii* are the causative agents of coccidiomycosis, known as Valley fever or Desert fever. Most infected individuals are asymptomatic and clear the infection with little to no medical intervention. Symptomatic disease in about 40% of infections presents across a clinical spectrum from acute pneumonia to chronic lung nodules to disseminated disease [1]. Chronic coccidioidomycosis severely decreases quality of life, and there is no cure for this infection. Broad-spectrum antifungals are currently the only treatment for chronic disease and induce severe side effects such as nausea, skin lesions, hair loss, and nervous system complications [1,2]. *Coccidioides* infections are on the rise, underreported, and often misdiagnosed [3].

Immunity to *Coccidioides* requires T helper (Th)1 and Th17 cellular responses that are trained by early innate recognition of *Coccidioides* in the lung [4,5]. Monocyte/macrophages and dendritic cells (DC) phagocytose pathogens and stimulate adaptive immunity via antigen presentation, cytokine and chemokine secretion and costimulatory signals, shaping the inflammatory and effector response. Macrophages and dendritic cells polarize into different subtypes in response to the tissue-specific signals and pathogen type present. Pro-inflammatory M1 macrophages induce tissue inflammation and cellular entry into infection sites, are associated with heightened phagocytic and clearance capabilities, and are typically fungicidal [6]. M2 macrophages are associated with post-inflammation wound repair and suppress inflammation by secreting IL-10 and arginase [7]. Tissue resident alveolar macrophages are sentinels and guardians of the immune microenvironment within the lung. The lung microenvironment maintains balance within the delicate airway spaces and alveolar sacs even during high inflammation [8]. Alveolar macrophages have high phagocytotic capabilities but require a high threshold of activation by pro-inflammatory signals. Macrophages have antigen presenting capabilities, but DCs are the professional antigen presenting cells dominantly responsible for activating and educating adaptive immune responses. Much like macrophages, DCs polarize into specialized subtypes in response to different pathogens.

DCs bridge the innate and adaptive immune response by bringing antigen, costimulation and cytokine signals to naïve T cells within secondary lymphoid organs to induce T cell activation and differentiation [9]. DCs loaded with *Coccidioides* antigen confer protection against virulent *Coccidioides* infection in susceptible mice [10]. CD4 T helper subtype differentiation is shaped by surface interactions with DCs and DC-secreted cytokines [11]. Th1/Th17 responses are typically induced by DC1s, while DC2s promote Th2 activation [11,12].

We examine macrophage and DC differentiation and functionality in response to *Coccidioides* to understand how these cells interact with this fungal pathogen. Using in vitro infection, we show that macrophages phagocytose *Coccidioides* arthroconidia poorly; monocytes respond and activate into macrophages (M0), but do not polarize into M1 or M2 subtypes nor upregulate activation markers. DCs polarize into DC1 in response to *Coccidioides* and upregulate maturation and costimulatory proteins. Multiple bacterial and fungal infections have been observed to increase alveolar macrophage and dendritic cell numbers in the lung [13,14]. Our infected lung image scoring reveals a spike in neutrophil counts at day 1 post-infection. In vivo DC1 and DC2 increase in frequency by day 7 post-infection within the lungs. Lastly, complete blood counts identify a spike in white blood cells and a drop in neutrophils at day 1 post-infection before returning to normal by day 7, suggesting that adaptive and immune cell responses mobilize in the peripheral blood following *Coccidioides* infection. Altogether, our data characterize early innate immune skewing of macrophages and DCs during *Coccidioides* infection and suggests that *Coccidioides* may actively inhibit macrophage and DC responses.

## 2. Materials and Methods

### 2.1. Mice

Six- to eight-week-old C57BL/6 male and female mice (JAX # 000664, The Jackson Laboratories, Bar Harbor, ME, USA) were utilized for experiments and sex matched whenever possible. Mice were housed and bred within the University of California Merced specific-pathogen free animal facility in compliance with the Department of Animal Research Services and approved by the Institutional Animal Care and Use Committee (protocol AUP18-000 approved 25 April 2018 and protocol AUP21-0004 approved 22 April 2021). 

### 2.2. Fungal Strain and Culturing for Method Arthroconidia Harvest

NR-166 avirulent *Coccidioides posadasii* (*Δcts2/Δard1/Δcts3*) laboratory strain derived from parent isolate C735 was used for all infections (BEI Resources, Manassas, VA, USA) [15]. Liquid 2x Glucose 1x Yeast Extract (2x GYE) media (Fisher Scientific, Hampton, NH, USA) was inoculated with frozen fungal stock and cultured at 30 °C in a shaking incubator for 72 h. Liquid culture was streaked onto 2x GYE agar plates and grown to confluency, then desiccated until the agar condensed. To obtain arthroconidia, the fuzzy white growth was scraped off the plate into PBS and filtered through a 40 µM mesh filter. The fungus was vortexed for 1 min to disassociate and centrifuged at 9000× *g* for 30 min at room temperature. The fungal pellet was washed with PBS and the pellet resuspended to the appropriate concentration in PBS for use. Arthroconidia suspension was stored at 4 °C for up to 3 months. Complete protocol used was from Mead et al. [16]. 

### 2.3. Calcofluor White Labeling of Coccidioides 

Powdered calcofluor white (Fluorescent Brightener: CFW; Sigma-Aldrich, St. Louis, MO, USA) was reconstituted in PBS at 5 mg/mL. Before each assay, arthroconidia were stained at 5 µg/mL in CFW for 5 min in the dark then washed twice in PBS. CFW-labeled arthroconidia suspensions were stored at 4 °C in the dark until use and any excess discarded [17]. 

### 2.4. Cell Line and Culture Conditions

RAW 264.7 mouse macrophage and NR8383 rat alveolar macrophage cell lines were used in the phagocytosis experiments. Bone marrow-derived cells were used in the macrophage and DC polarization experiments. Cells were cultured in phenol red-free DMEM 10% FBS 1% penicillin/streptomycin 1% l-glutamine (DMEM complete media) for macrophages or RPMI 10% FBS 1% penicillin/streptomycin 1% l-glutamine (RPMI complete media) for monocytes. RAW 264.7 were provided by Anita Sil at University of California San Francisco and NR8383 by Laurent Dejean at California State University Fresno. 

### 2.5. Phagocytosis Assay and Data Acquisition 

5 × 10^5^ cells were plated in 1 mL per well in a 12 well plate. Lipopolysaccharide (LPS: Millipore Sigma-Aldrich, St. Louis, MO, USA) stimulation was used as a positive control at 5 ng/mL final concentration. CFW-labeled arthroconidia were plated with cells at a 1:1 ratio. Two duplicate plates were created, one incubated at 37 °C and one incubated at 4 °C (negative phagocytosis control). Following a 2 h incubation phagocytosis was halted by putting the plates on ice to prepare for imaging. For adherent cells, supernatant was aspirated from the plates. For non-adherent cells, the plates were centrifuged at 1200× rpm for 5 min in 10 °C. To quench the fluorescence of externally bound *Coccidioides*, each well was washed with a solution of Congo red (Fisher Scientific, San Jose, CA, USA) in PBS at a concentration of 5 mg/mL for 1 min. PBS was added to the plate to dilute the quench and supernatant removed. Each well was replenished with 1 mL of complete media for imaging. In each plate, controls with unquenched wells (no Congo red wash) were included. Cells were analyzed via imaging and flow cytometry. 

### 2.6. Phagocytosis Imaging 

Plates were incubated on ice for 20 min to halt the phagocytosis process on the 37 °C plate while imaging the 4 °C plate. Plates were imaged at 20× using the Invitrogen™ EVOS™ FL Digital Inverted Fluorescence Microscope (ThermoFisher, Waltham, MA, USA). Each well was imaged using a cross pattern (top, center, left, right, bottom) for consistency. Scoring criterion was as follows: macrophages in contact with at least one or more *Coccidioides* were counted as participating in an association event; macrophages with internalized *Coccidioides* were counted as participating in a phagocytosis event. Macrophages participating in both events were counted for both events. Dead, out of frame, out of plane macrophages were not included in the total cell count for each image. Phagocytosis and association frequency was determined by (total # of macrophages participating in each event)/(total live cells in each image). 

### 2.7. Antibody Staining and Flow Cytometry 

Mouse pulmonary draining lymph nodes (dLN) and lungs were mechanically homogenized and collected in a PBS/1% FBS solution and filtered through a 100 μM mesh filter. Cell suspensions were centrifuged at 1200× rpm for 5 min at 10 °C. Red blood cells were lysed in 1× lysis buffer made from 10× Ammonium Chloride Lysis Buffer Stock (NH_4_Cl (ammonium chloride) 8.02 gm NaHCO_3_ (sodium bicarbonate) 0.84 gm EDTA (disodium) 0.37 gm in 500 mL Millipore water; (Fisher Scientific, San Jose, CA, USA), washed, and resuspended in PBS/1% FBS. Cells were resuspended at 2 × 10^6^ cells and stained at 50 µL (using antibodies described below from eBioscience (San Diego, CA, USA) unless otherwise noted) for 30 min in the dark at 4 °C. Cells were washed with staining media and fixed for 45 min in the dark at room temperature, washed and resuspended in 100 μL PBS/1%FBS for flow cytometry acquisition. Cells were stained with Fixable Viability Dye eFluor 506 (1:500), anti-CD8α Pe-Cy7 (clone 53–6.7; 1:400), anti-CD11c FITC (clone HL3, BD Biosciences; 1:400), anti-F4/80 PerCP-Cy5.5 (clone BM8; 1:400), anti-MHC-II (I-A/I-E) APC-Cy7 (clone M5/114.15.2, BioLegend, 1:200), anti-CD86-PE (clone PO3.1, 1:200), anti-SIRPα/CD47 APC (clone P84, BioLegend, 1:400), anti-CD38 APC (clone 90; 1:200), and anti-CD206 Pe-Cy7 (Clone MR6F3; 1:400). Data was acquired on a LSRII (BD) and analyzed using FCS Express Version 4 and 7 Research Edition (DeNovo Software, Pasadena, CA, USA). 

### 2.8. Bone Marrow Harvest for Macrophage and DC Polarization Experiments 

Bone marrow was harvested from femurs and pooled according to sex. Bones were mechanically crushed using mortar and pestle and washed with RPMI complete media. Cells were filtered using a mesh 100 μM filter and centrifuged at 1200× rpm for 5 min at 10 °C. Red blood cells were lysed for 1 min, washed with PBS, resuspended in complete media, and counted. 

### 2.9. Macrophage Polarization 

Bone marrow-derived monocytes were plated in RPMI complete media in tissue culture treated plates with phorbol 12-myristate 13-acetate (PMA) (Fisher Scientific, San Jose, CA, USA) at a final concentration of 1 μg/mL. After 24 h, non-adherent cells were collected and disposed. Adherent cells were scraped into fresh media resuspended at 1 × 10^6^ cells/mL in RPMI complete media and replated in a new 12 well plate. The conditions were as follows: no stimulation, 100 ng/mL LPS, 20 ng/mL IL-4, *Coccidioides* arthroconidia at 1:1 ratio to cells, *Coccidioides* plus LPS, and *Coccidioides* plus IL-4. Plated cells were incubated for 24 h at 37 °C then all cells, adherent and non-adherent, were harvested for flow cytometry (See Supplemental Figure S4 for gating strategies). 

### 2.10. Dendritic Cell Polarization

30 × 10^6^ bone marrow-derived cells were plated in 30 mL of RPMI complete media with final concentration 20 ng/mL GM-CSF on 100 mm non-tissue culture treated plates [18]. On day 3, 15–30 mL of fresh media and GM-CSF was added to each plate. On day 6, non-adherent cells were collected by harvesting the supernatant, incubating plates with 5 mL of 3 mM EDTA/PBS for 1–2 min, washing with media and collecting cells. Cells were centrifuged for 7 min at 1000× rpm at 10 °C. Cells were resuspended in 20 mL RPMI complete media at appropriate concentration for experimental usage. A total of 2 × 10^6^ cells per 2 mL were plated in a 6 well plate and 0.5 ng/mL IL-4 added to the DC2 condition wells. On day 7, 1 µg/mL LPS was added to DC1 condition wells. On day 8, cells were stimulated with or without *Coccidioides* plus LPS or IL-4 as in the macrophage polarization assays above. Cells were incubated for 48 h at 37 °C and harvested for flow cytometry (See Supplemental Figure S5 for gating strategies).

### 2.11. Dendritic Cell Polarization with Supernatant Assay

Following the methods outlined in Section 2.10 above, BMDCs were prepared, and supernatant was harvested from the wells for cell stimulation in place of direct stimulation. On day 10 post stimulation, plates were spun down at 1200× rpm for 5 min at room temperature to minimize harvesting cells. Supernatant was collected, passed through a sterile 40 μM mesh cell strainer (Fisher Scientific, San Jose, CA, USA), and then syringe filtered using a 0.45 μM filter unit (Merck Millipore, Burlington, MA, USA). Then, 2 mL media from each condition was added to fresh BMDCs on day 8 for stimulation. Cells were incubated for 48 h at 37 °C and harvested for flow cytometry. 

### 2.12. In Vivo Infection and Tissue Harvest

Mice were intranasally infected by dotting arthroconidia suspended in PBS onto their nostrils and waiting for inhalation before repeating for the entire experimental dose. Experimental conditions were as follows: uninfected, 30 μL PBS mock infection, and 10^5^ arthroconidia in 30 μL PBS. Mice were euthanized on day 1 or 7 post-infection for tissue harvest and analysis. Tissues collected were as follows: peripheral blood was collected for complete blood count analysis, whole lung for flow cytometry and immunohistochemistry, lung draining lymph node and spleen for flow cytometry analysis. 

### 2.13. Complete Blood Count

50–100 μL blood was collected via retro-orbital bleeding in BD Biosciences K2E Microtainer tubes (K2EDTA) (BD Biosciences Pharmingen, San Diego, CA, USA) and analyzed on the Drew Scientific Hemavet 950 (Drew Scientific, Erba Diagnostics, Miami Lakes, FL, USA). 

### 2.14. Immunohistochemistry, Imaging, and Analysis 

Left lung lobes were embedded in optimal cutting temperature (OCT) compound (Leica Biosystems, Wetzlar, Germany) for histological analysis. Lung samples were tissue sectioned at 10 μM using a Leica CM1860 cryostat and were immediately fixed in ice-cold acetone. Samples were washed with PBS, 1% and 5% blocking buffer (PBS and BSA) at room temperature. Sections were stained with anti-CD11c FITC (eBioscience, San Diego, CA, USA, clone N418, 1:500), anti-Ly-6G Alexa Fluor 700 (BioLegend, San Diego, CA, USA, clone 1A8, 1:50), anti-Siglec F/CD170 PE (BD, clone E50-2440, 1:50), and either anti-F4/80 PE-Dazzle^TM^ 594 (BioLegend, clone BM8, 1:250) or anti-EpCAM/CD326 Alexa Fluor 594 (BioLegend, clone G8.8, 1:500) for 2 h at room temperature then washed and imaged. A Zeiss LSM 880 confocal microscope at 10× (10×/0.45 Plan Apochromat; 420640-9900) and 40× (40×/1.2 LC LCI Plan Apochromat; 420862-9970-799) objectives was used for imaging. Each lung section was imaged in three consistent sections across all mice and conditions: top, middle, and bottom of lobe. Counts from all lobes were combined to give a final total count for each lung. Lung images were blinded and scored by two independent scorers via the criterion guide below. Intermediate monocytes (iMO) were identified as F4/80+SiglecF-Ly6G+, alveolar macrophages as CD11c+SiglecF+F4/80+, neutrophils as F4/80-Ly6G+, dendritic cells as CD11c+.

### 2.15. Statistics

Experimental data were analyzed using paired Student’s *t*-test and all data analyzed for outliers using Grubbs Outlier exclusion analysis with GraphPad Prism v.8 for Windows Software (GraphPad Software, San Diego, CA, USA). Figure legends denote what comparisons took place, if outliers were detected and excluded, and the *p*-value for each figure.

## 3. Results

### 3.1. Poor Coccidioides Phagocytosis by Monocytes and Macrophages

**Figure 1:**
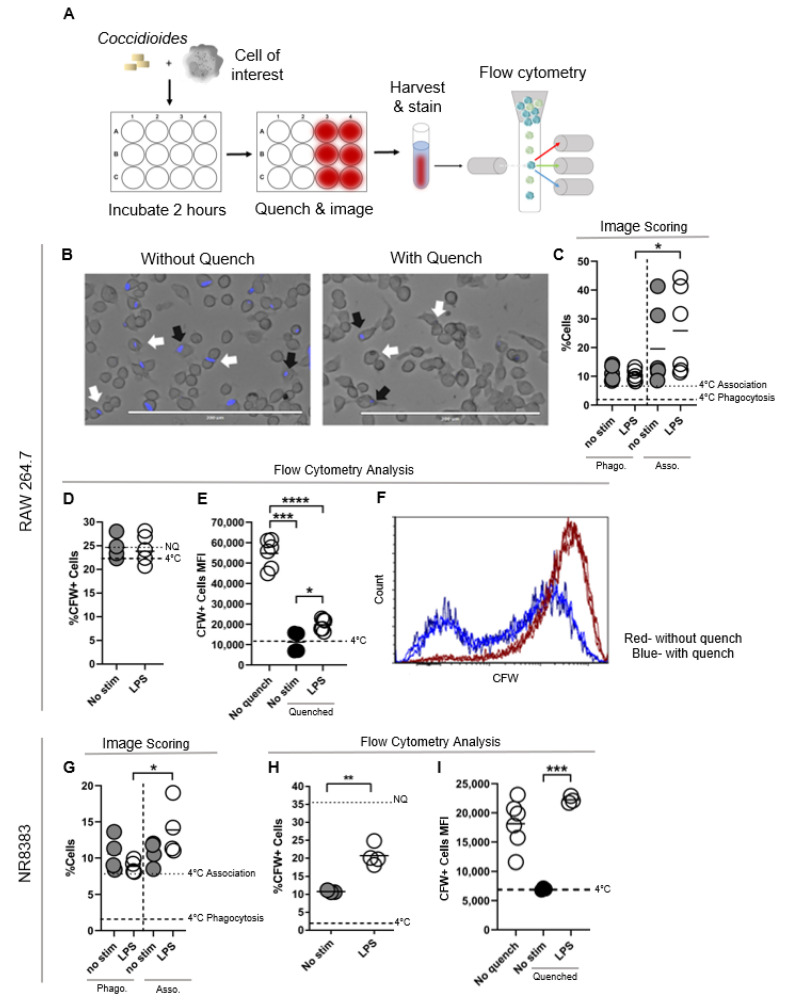
Macrophages poorly phagocytose *Coccidioides*, although mouse macrophages have a stronger association with *Coccidioides*. Phagocytosis analysis via flow cytometry complements traditional imaging assay while increasing output efficiency and allowing analysis of association versus phagocytosis. (**A**) 5 × 10^5^ cells were incubated 1:1 with CFW labeled *Coccidioides* for 2 h then left unquenched or quenched using Congo red dye. Cells were imaged then harvested and processed for flow cytometric analysis. (**B**–**F**) Data from RAW 264.7 mouse macrophages. (**G**–**I**) Data from NR8383 rat alveolar macrophages. (**B**) Bright field image of CFW-*Coccidioides* infected RAW 264.7 cells. Externally bound *Coccidioides* is quenched by Congo red, leaving only the internalized *Coccidioides* visually discernable by the Pacific blue marker for imaging by fluorescence labeling. White arrows indicate internalized (phagocytosed) *Coccidioides,* and black arrows show externally bound or externally associated *Coccidioides* in un-quenched and quenched conditions. (**C**–**I**) Non-quenched controls = cells harvested but un-quenched, No stim = *Coccidioides* only conditions without additional stimulation, LPS = pro-inflammatory stimulant, 4 °C = negative phagocytosis control. (**D**,**H**) Frequency of CFW+ cells gated from Singlet/Live cells. (**E**,**I**) Mean fluorescence intensity (MFI) of the cells from CFW+ population. (**F**) Representative histogram of CFW fluorescence in RAW 264.7 macrophages incubated with CFW-labeled *Coccidioides* at 37 °C under quenched conditions (blue line) indicating *Coccidioides*, or non-quenched (red line) indicating *Coccidioides* association and engulfment. (**C**,**G**) Image scoring data. Left side: frequency of cells with internalized *Coccidioides*; right side: frequency of cells associating with *Coccidioides* externally. *n* = 3–8, data are representative from 2–3 experiments. For all plots, line indicates mean, and each dot is one experimental replicate. Data was analyzed using an unpaired Student’s *t*-test and outliers excluded using Grubbs Outlier exclusion analysis. * *p* < 0.05, ** *p* < 0.005, *** *p* < 0.0005, **** *p* < 0.00005.

To understand macrophage responses to *Coccidioides*, we first assessed macrophage phagocytic function against *Coccidioides* using traditional image scoring methods and a novel flow cytometry approach. Traditional methods for phagocytosis assays require imaging and independent, blinded scoring which are time-intensive and require tight scoring guidelines to ensure consistency and prevent bias between samples. We adapted a flow cytometry analysis-based approach for analyzing phagocytosis by utilizing fluorescently labeled *Coccidioides* and quenching externally bound *Coccidioides* with Congo red dye that binds amyloid proteins (Figure 1A). 

This method increases the speed and output for phagocytosis analysis, allowing interrogation of multiple cells and conditions simultaneously. We first tested a mouse macrophage cell line, RAW 264.7, using these methods (Figure 1B–F). Representative images of RAW 264.7 cells incubated 1:1 with CFW-labeled *Coccidioides* at 37 °C for two hours show examples of fungal arthroconidia externally bound or closely associated with *Coccidioides* (Figure 1B, white arrows) or internalized (Figure 1B, black arrows). Fungal association refers to cell events where *Coccidioides* is in direct contact with a cell but without clear internalization, while phagocytosis is defined as fungal internalization (Figure 1C). After imaging, cells were harvested and analyzed using flow cytometry (Figure 1D–F,H,I). Without fluorescent quenching of externally bound *Coccidioides*, a representative histogram shows most mouse macrophages interacting with *Coccidioides*, masking our ability to define fungal phagocytosis (Figure 1F, red histogram). However, Congo red quenching results in a bimodal macrophage population, with CFW+ macrophages that have internalized *Coccidioides* and CFW− macrophages bound to *Coccidioides* on the surface (Figure 1F, blue histogram). While on average 19.57% of mouse macrophages interact with *Coccidioides*, only 10.85% successfully internalize *Coccidioides* within two hours (Figure 1C, phagocytosis left column, association right column). With LPS stimulation, 25.83% of macrophages interact with *Coccidioides* but only 10.24% successfully internalize *Coccidioides*. 

Thus, although most of these macrophages are associated with *Coccidioides*, few mouse macrophages are successfully phagocytosing *Coccidioides*, even with the addition of a strong stimuli, LPS. We next examined a rat alveolar macrophage cell line, NR8383 where we observed enhanced phagocytosis in the presence of LPS (Figure 1G–I). On average, 10.7% of the raw alveolar macrophages associated with *Coccidioides* and 10.5% phagocytose successfully by confocal fluorescent imaging. With LPS stimulation, 13.9% rat alveolar macrophages associated with *Coccidioides* and 8.9% participated in successful phagocytosis. The interaction to phagocytosis frequency gap is smaller with rat alveolar macrophages (13.9% to 8.9%; 1.6-fold reduction) than mouse macrophages (25.83% to 10.24%; 2.5-fold reduction). However, there was no significance between unstimulated and LPS stimulated alveolar macrophages (Figure 1I, left column). 

These differences between the mean fluorescence intensity (MFI) flow data and traditional scoring data can be explained by the fact that the scoring method criterion counts the number of cells that participate in each type of interaction with *Coccidioides* out of all cells within the image frame, while flow cytometry allows for detailed recording of fluorescent intensity as a metric of how many fungi a single cell interacts with. Our phagocytosis data reproduce previous findings for macrophages, where pro-inflammatory stimuli enhance phagocytotic mechanisms [19]. We observed that rat and mouse macrophages poorly phagocytose *Coccidioides* with higher surface association than phagocytosis, but that mouse macrophage association occurs more frequently than rat alveolar macrophage association. 

### 3.2. Coccidioides Blocks Monocytes in a Poorly Activated, Non-Differentiated State

**Figure 2:**
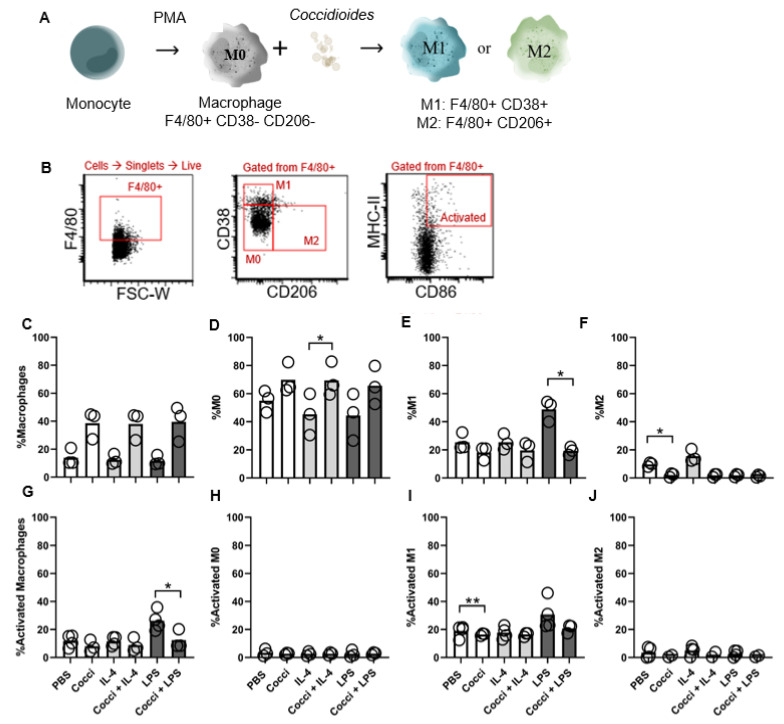
Monocytes activate into macrophage in response to *Coccidioides* but do not polarize into M1/M2 subtypes and largely lack activation/maturation markers. (**A**) Schematic illustrates monocytes differentiation into M0 and polarized M1/M2 potential outcomes with *Coccidioides*. PMA (phorbol 12-myristate 13-acetate), an activator of protein kinase C (PKC), was used as a positive stimulant for M0. LPS (100 ng/mL) acted as M1 positive control and IL-4 (20 ng/mL) as M2 positive control. Cocci = *Coccidioides* added at 1:1 ratio with cells. (**B**) Representative flow plots show the gating strategy for analysis; sample shown was stimulated with *Coccidioides*. See Supplemental Figure S4 for complete gating strategy. (**C**,**G**) macrophages are defined as all F4/80+ cells, (**D**,**H**) M0 defined as CD11b-F4/80+, (**E**,**I**) M1 as CD38+ F4/80+, and (**F**,**J**) M2 as CD206+ F4/80+. (**G**–**J**) Activated cells are defined as CD86+MHC-II+ and are gated off their respective population. *n* = 3–4, data representative of 3 experiments. The bar within each data group indicates the mean, each individual circle represents one biological replicate averaged from all technical replicates within each experiment. Statistics show comparisons between *Coccidioides* and non-*Coccidioides* conditions. Data was analyzed using unpaired Student’s *t*-test. * *p* < 0.05, ** *p* < 0.005.

We next sought to characterize monocyte differentiation into macrophage subtypes and activation state in response to *Coccidioides*. Immune cell polarization and activation provides targeted pathogen control during early immune response, laying the foundation for adaptive immunity. Bone marrow-derived monocytes were cultured with polarizing stimulants and *Coccidioides*, and polarization assessed by surface protein expression. PMA induces M0 differentiation, LPS stimulates M1 differentiation, and IL-4 promotes M2 differentiation (Figure 2A). Representative flow cytometry plots and gating strategy are shown for *Coccidioides* stimulated monocytes in Figure 2B. On average, 13.9% of monocytes differentiated into macrophages (all F4/80+ cells) in response to PMA stimulation (written as PBS control stimulation in Figure 2C). *Coccidioides* tends to promote macrophage differentiation in BMDMs. Similarly, in the presence of *Coccidioides*, more monocytes trended towards differentiation into M0 macrophages (69.9% with *Coccidioides*, 69.4% for *Coccidioides* plus IL-4 and 65.6% with *Coccidoides* plus LPS), but poorly differentiated into M1 and M2 macrophages (Figure 2D–F). While the presence of *Coccidioides* seems to induce monocytes to differentiate into M0 macrophages, *Coccidioides* also appears to block M1 and M2 polarization (Figure 2E,F). M1 differentiation decreases in the presence of *Coccidioides* with LPS relative to LPS alone (48.76% to 19.31% (Figure 2E)). Additionally, exposure to *Coccidioides* reduces CD86 and MHC-II co-expression, even in the presence of LPS across all macrophage and polarized subtypes, dropping the activated M1 frequency from 30.67% to 20.77% (*p* = 0.1882) (Figure 2G–J). Overall, these data suggest low macrophage activation and maturation in the presence of *Coccidioides*. 

### 3.3. DCs Favor DC1 Polarization in Response to Coccidioides but Lack Activation and Maturation Markers

**Figure 3:**
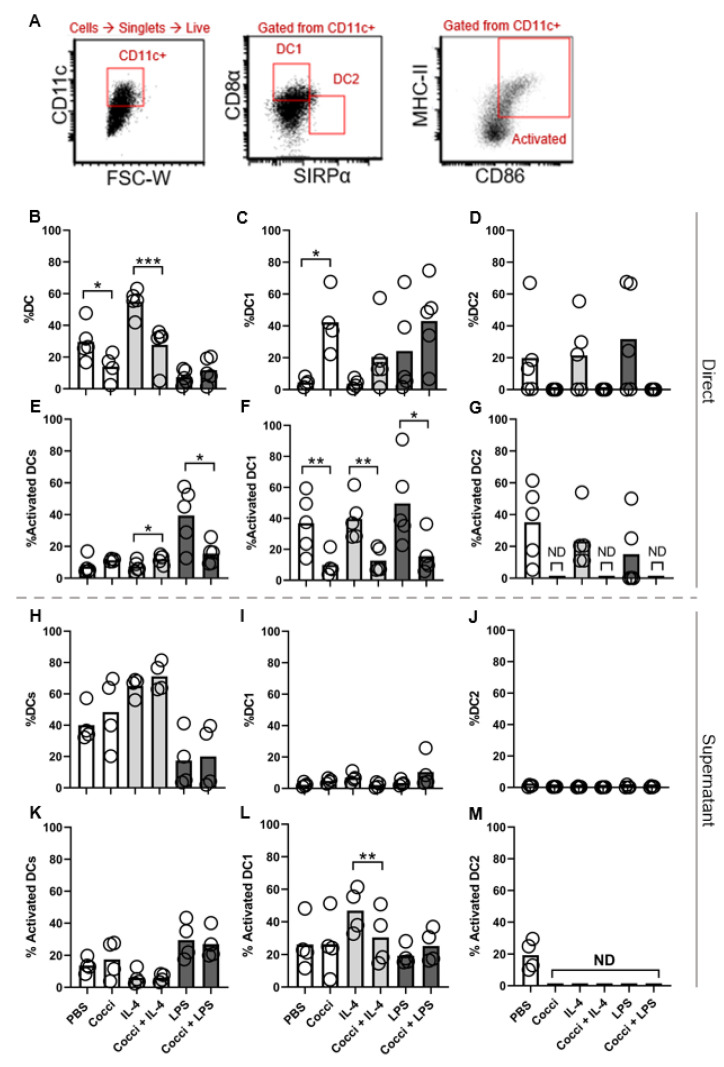
DCs polarize to DC1 in response to *Coccidioides* but do not upregulate CD86 and MHC-II expression. (**A**) Representative flow plots show gating strategy used for analysis; sample shown is stimulated with *Coccidioides*. See Supplemental Figure S5 for complete gating strategy. Using a C57BL/6 bone marrow-derived DC culture system to generate DCs, we assessed DC polarization. Unstim refers to DC culture conditions without cytokine stimulation. LPS (1 µg/mL) was added as a DC1 control and IL-4 (0.5 ng/mL) as a DC2 control. *Coccidioides* was added in a 1:1 ratio with cells. (**B**–**G**) DCs were directly stimulated with *Coccidioides*, LPS and/or IL-4. (**H**–**M**) DCs were stimulated using supernatants generated in (**B**–**G**). (**B**,**H**) DC frequency of all CD11c+ cells including DC1 and DC2. (**C**,**D**,**I**,**J**) DC1 and DC2 populations were gated from total CD11c+ population. (**C**,**F**,**I**,**L**) DC1 are defined as CD8α+SIRPα-CD11c+ and (**D**,**G**) DC2 as CD8a-SIRPa+CD11c+. (**E**–**G**,**K**–**M**) Activated cells are defined as CD86+MHC-II+; activated cells are gated from their respective subtype population. ND = not determined due to lack of cells from previous gate. *n* = 4–5, data are representative of 5 experiments for direct assay, and 3 experiments for supernatant assay. The bar within each data group indicates the mean, each individual circle represents one biological replicate averaged from all technical replicates within each experiment. Statistics show comparisons between *Coccidioides* and non-*Coccidioides* conditions. Data was analyzed using unpaired Student’s *t*-test and outliers excluded using Grubbs Outlier exclusion analysis * *p* < 0.05, ** *p* < 0.005. *** *p* < 0.0005.

We next interrogated DCs to determine if there is a similar polarization and maturation block as is found in macrophages by *Coccidioides*. We cultured bone marrow-derived DCs to evaluate DC maturation and polarization in the presence of *Coccidioides*; representative flow plot shows the gating strategy for DCs (Figure 3A). We observed a block in total CD11c+ DC frequency in the presence of *Coccidioides* (Figure 3B). IL-4 treatment induces DC maturation and induced the highest total CD11c+ frequency (Figure 3B). The addition of *Coccidioides* to IL-4 culture resulted in a significant reduction in CD11c+ cell differentiation from an average of 54.91% to 27.75% (Figure 3B). In the CD11c+ population, all stimulation conditions in the absence of *Coccidioides* induced both DC1 and DC2 differentiation (Figure 3C,D). However, when exposed to *Coccidioides*, only non-polarized and DC1 (CD8α+SIRPα-) differentiation occurred, with DC1 frequency increasing from 4.52% in PBS condition to 42.21% in the presence of *Coccidioides* (Figure 3C). Though not statistically significant (*p* = 0.0645), DC frequency increased from 24.25% with LPS to 43.05% with LPS and *Coccidioides*. Further, little to no DC2 polarization occurred in the presence of *Coccidioides* under any stimulation conditions (Figure 3D).

Strikingly, when in the presence of *Coccidioides*, DCs did not differentiate into DC2, with DC2 frequency dropping from an average of 19.84% in PBS to 0.03% in the presence of *Coccidioides* (Figure 3D). Further, DC1s cultured with *Coccidioides* expressed MHC-II and CD86 at a significantly lower frequency indicating reduced maturation and activation capacity in the presence of *Coccidioides* even when co-cultured with LPS (Figure 3E–G). Total DC activation frequency when stimulated with LPS dropped from 39.34%without *Coccidioides* to 14.49% with *Coccidioides* (Figure 3E). These data suggest that *Coccidioides* inhibits DC maturation, activation and DC2 polarization. 

Next, we sought to characterize whether this inhibition was contact dependent. We performed an indirect stimulation assay using supernatant from the direct stimulation assay in Figure 3B–G. Supernatants from PBS, LPS and IL-4 induced similar CD11c+ cells as found in the direct assay. However, the supernatants from the *Coccidioides* treated BMDC did not inhibit DC generation. Unlike the direct polarization assay where the addition of *Coccidioides* caused a significant reduction in DC and DC1 activation, there was no significant change in the supernatant polarization assay (Figure 3K,L). There was significant reduction in the amount of activated DC1 between IL-4 +/− *Coccidioides* indicating some activation blockage by secreted factors under this condition (Figure 3L). Further, we observed a reduction in DC1 frequency when BMDCs were incubated with supernatant relative to arthroconidia, with DC1 frequencies averaging 42.21% in the direct assay and 4.77% in the supernatant assay (Figure 3C and Figure 3I, respectively). These data suggest that DC responses are largely a result of direct contact with *Coccidioides*. 

### 3.4. Lung Immunohistochemistry Reveals Increased Neutrophil Frequency Post-Infection

**Figure 4:**
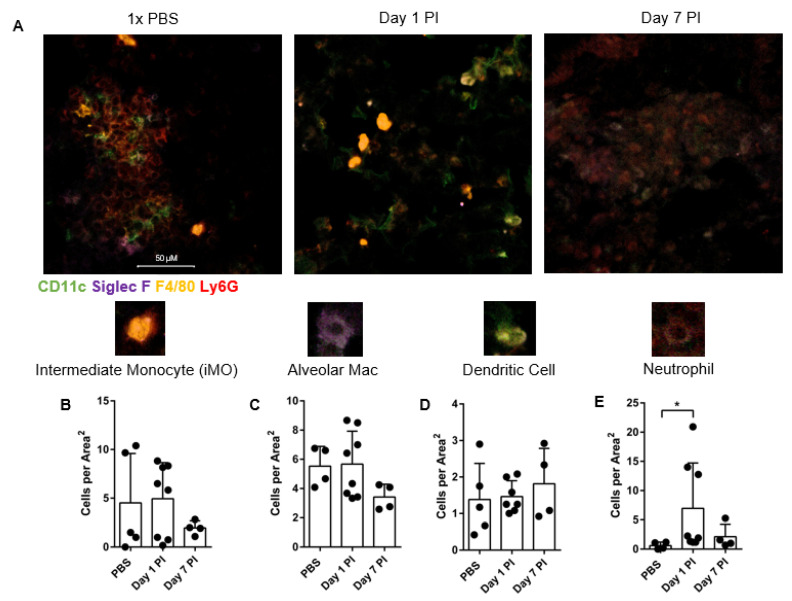
Immunohistochemistry and lung image scoring reveals heightened CD11c positive and Ly6G positive cells in the lung post infection. Lungs were processed at day 1 and day 7 post infection (PI) and fresh-frozen. (**A**) In the representative plot, PBS is the mock infection whereas Day 1 and Day 7 show 10^5^ arthroconidia intranasal infection at 40× confocal imaging. 50 μM scale bar applies to all images. (**B**) Intermediate monocytes (iMO) are defined as F4/80+ Ly6G+, (**C**) alveolar macrophages (Mac) as F4/80+ CD11c+ Siglec-F+, (**D**) DCs as CD11c+ F4/80−, and (**E**) neutrophils as Ly6G+ F4/80− CD11c−. Images scored are from 40× magnification. Each image represents 742,819.8969 uM^2^ in Area^2^, calculated from square image of 862 × 862 μm^2^. Images were blind scored by two independent counters. For all plots displayed line indicates mean, and each dot is one experimental replicate for each lung sample averaged over 5 images and two blind-scorers. *n* = 4–7, representative of 4 experiments. Comparisons were made between mock PBS infection and infected at each time point. Data was analyzed using unpaired Student’s *t*-test and outliers excluded using Grubbs Outlier exclusion analysis; * *p* < 0.05.

To evaluate the immune responses in vivo, we intranasally infected mice with CFW-labeled *Coccidioides* and processed the lungs via immunohistochemistry to visualize immune cell quantity and localization patterns in the lung. Figure 4A shows representative lung images taken at 40× magnification of PBS mock infected lungs and *Coccidioides* infected lungs at day 1 and day 7 post *Coccidioides* infection. There were no significant changes in intermediate monocyte (F4/80+Ly6G+), alveolar macrophage (F4/80+Siglec-F+CD11c+), or DC (CD11c+F4/80−) counts between conditions (Figure 4B–D). 

Neutrophil (Ly6G+F4/80−) counts increased at day 1 post-infection by 11-fold compared to mock PBS infection, before returning to normal by day 7 post-infection (Figure 4E). The innate cell changes within the lung following fungal infection indicates an inflammatory immune response occurs. However, we were unable to directly ascertain localization patterns of immune cells relative to *Coccidioides* within the lung tissue. This is in part due to the vast tissue survey needed to determine this information and limitations in visualizing both immune cells and *Coccidioides* by immunohistochemistry at 10× (data not shown) and 40×. 

### 3.5. DC1 and DC2 Frequency Increase in Lungs Post Infection 

We next utilized flow cytometry to analyze lungs and lung-draining lymph nodes (dLN) following intranasal infection to assess DC numbers and functional markers in vivo. First, to confirm that a systemic cellular response occurs following avirulent *Coccidioides* infection, we evaluated peripheral blood cell numbers and frequencies using complete blood count analysis. White blood cell peripheral blood numbers increase on day 1 post-infection and drop below mock-infection levels by day 7 (Supplemental Figure S1A), suggesting that the host recognizes an infection and mounts a systemic response. Neutrophil peripheral blood numbers drop slightly day 1 post-infection before returning to mock infection levels at day 7 post-infection (Supplemental Figure S1C). 

**Figure 5:**
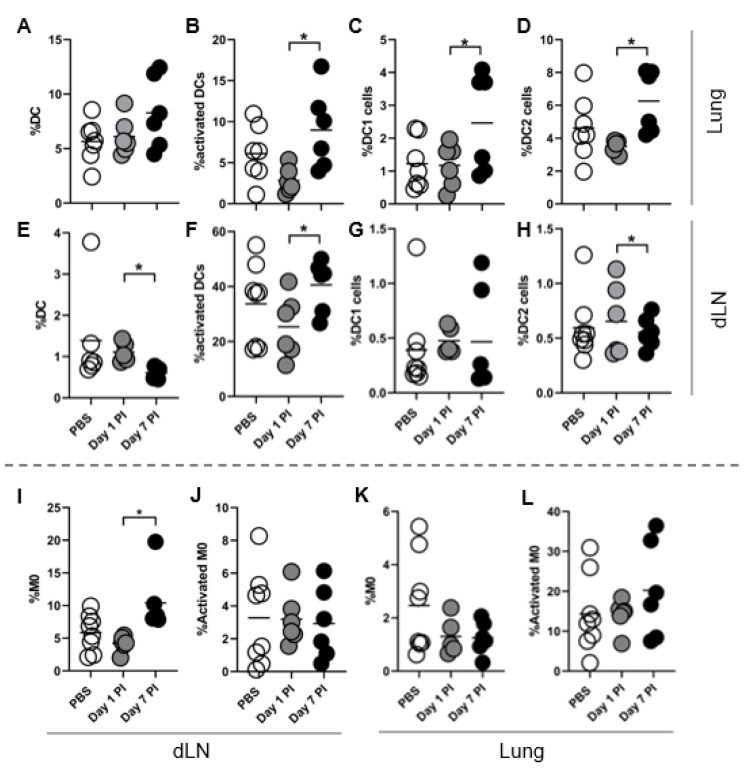
DC1 and DC2 increase in frequency at day 7 post-infection. C57BL/6 mice were intranasally infected with 10^5^ arthroconidia or PBS. Cell frequencies are comparable to uninfected mice (data not shown). Both lung lobes were collected and homogenized for flow cytometry analysis. Draining lymph node (dLN) refers to the pulmonary lung-draining LN. DC1 (**C**,**G**) and DC2 (**D**,**H**) populations are gated from CD11c+ population (**A**,**E**). DC1 are defined as CD8a+SIRPa-CD11c+ and DC2 as CD8a-SIRPa+CD11c+. (**B**,**F**) Activated DC frequency accounts for all DCs including DC/DC1/DC2. (**I**,**K**) Macrophages are defined as F4/80+ cells and (**J**,**L**) activated population is gated from the F4/80+ population. Activated cells are defined as CD86+MHC-II+ cells. *n* = 6–8 individual mice from 3 experiments. For all plots displayed line indicates mean and each dot is one experimental replicate. Comparisons were made between mock PBS infection and infected at each time point. Data was analyzed using unpaired Student’s *t*-test and outliers excluded using Grubbs Outlier exclusion analysis; * *p* < 0.05.

No changes are observed in peripheral blood monocyte or eosinophils during avirulent infection (Supplemental Figure S1B,D). CD11c+ cell frequency was normal to slightly elevated in the lung and slightly decreased in the lung-draining LN on day 7 post-infection as measured by flow cytometry (Figure 5A,E). The frequency of activated CD11c+ cells co-expressing CD86 and MHC-II was elevated in the draining LN and lungs on day 7 post-infection relative to day 1 post-infection, returning to mock PBS infected levels (Figure 5B,F).

In vitro bone-marrow derived DCs differentiated into DC1 but not DC2 subsets so we next assessed DC subtypes in the lung and draining LN following in vivo *Coccidioides* infection. DC1 and DC2 cell lung frequencies significantly expanded, but with considerable variability (Figure 5C,D). DC subset frequency was unchanged in the lung-draining lymph node except for a mild decrease in DC2 on day 7 post infection relative to day 1 (Figure 5G,H). We next assessed macrophage changes during infection. Macrophage frequency significantly increased in the lungs and tended to decrease in the draining LN at day 7 post-infection (Figure 5I,K). Activated macrophage frequency based on co-expression of MHC-II and CD86 was unchanged following infection (Figure 5J,L). Analysis of macrophages and DCs expressing only MHC-II or CD86+ DCs in the lung and draining LN revealed no significant differences across tissues or time post-infection, except for a decrease in CD86+ macrophage frequency in the draining LN at day 7 post-infection (Supplemental Figure S2B). Total DCs in the draining LN increased at day 1 post infection before dropping at day 7, with no total changes observed in activated DC numbers (Supplemental Figure S3A,B). Together these data indicate a mixed DC1 and DC2 differentiation response within the lung and mild activation of DCs in response to avirulent Coccidioides alone.

## 4. Discussion

In this study we applied flow cytometry and imaging techniques to study innate immune responses to *Coccidioides*. We demonstrate that murine macrophages poorly phagocytose *Coccidioides*, while rat alveolar macrophages have higher phagocytosis rates. This corresponds to previous work done with macrophages and general observations regarding the high phagocytosis ability of alveolar macrophage [7,19]. We further found that *Coccidioides* induces efficient monocyte differentiation into macrophages, but largely blocks differentiation at the M0 stage, preventing polarization into M2 macrophages, reducing M1 differentiation and decreasing macrophage activation even in the presence of strong activating and differentiating stimuli. *Coccidioides* also poorly activates DCs, inhibiting DC activation by LPS and IL-4. Bone marrow derived DCs preferentially polarize into DC1 with no DC2 differentiation and poorly activate in vitro in response to *Coccidioides*. In contrast, in vivo DCs and macrophages show an increased activation frequency in response to avirulent *Coccidioides* post infection, although activation frequency is rather low, and DCs show a mixed DC1/DC2 recruitment in the lungs. Peripheral blood analysis reveals no changes in blood monocyte counts over infection (Supplemental Figure S1). Altogether, these data suggest innate immune cells respond and recognize *Coccidioides* but there may be undefined virulence mechanisms allowing fungal escape from phagocytosis and impairment of innate immune cell polarization and immune activation in vitro.

Macrophages are typically associated with pathogenic responses for microbial and foreign body clearance. Their ability to polarize into M1 or M2 subtypes allows for targeted, tailored responses. Proinflammatory cytokines and reactive oxygen species are associated with classically activated M1 macrophages with antimicrobial activity [20]. Anti-inflammatory and wound-repairing signals are associated with alternatively activated M2 macrophages with tissue repair properties. Reprogramming of activated M0 macrophages into M1 or alternatively activated M2 macrophages is reinforced by the secreted cytokines and metabolites produced by the developing population. *Coccidioides* secreted factors suppress nitric oxide and inducible nitric oxide synthase by bone marrow-derived macrophages, and similar factors may regulate macrophage polarization [21]. Production of oxide species (OS) is associated with pro-inflammatory M1 subset; however, with *Coccidioides*, iNOS production is not essential for phagocytosis or fungicidal killing. This suggests that *Coccidioides* suppression of OS production in macrophages could be both a functional inhibition as well as specialization inhibition.

The monocyte to macrophage differentiation in response to *Coccidioides* infection, although not previously measured, is consistent with prior studies that found high IL-6, IL-12, TNF and MIP-2 production by *Coccidioides*-infected peritoneal macrophages [22]. Most surprising was the blocking phenomena where macrophages co-cultured with *Coccidioides* and LPS failed to upregulate MHC-II and CD86 co-expression. *Coccidioides* spherules express metalloproteinase 1 (MEP1) which cleaves the spherule outer wall glycoprotein (SOWgp) antigens, decreasing chances of innate immune cell detection via pattern recognition receptors [23]. Our studies utilize *Coccidioides* in the arthroconidia phase, the soil morphology that does not express MEP1, which suggests that *Coccidioides* may have previously undefined virulence mechanisms that block MHC-II and CD86 upregulation. In *Cryptococcus neoformans* infection, productive immune responses within lung macrophages induce heightened iNOS mRNA levels and M1 macrophages inhibit fungal growth more effectively than M2 macrophages [24]. *Paracoccidioides brasiliensis* antigen stimulates strong M1 macrophage polarization within mouse peritoneal-derived macrophages and in vivo studies show M1 macrophages are more critical for fungal clearance than M2 [25,26]. The specialization block induced by *Coccidioides* presence in our in vitro culture suggests a possible virulence mechanism where macrophage polarization to M1 is inhibited as means of inhibiting host activation of the specialized, pro-inflammatory macrophage function.

In vitro phagocytosis of *Coccidioides* by macrophages is known to be weak. However, our data suggest alveolar macrophages may be more vital than migratory monocytes for *Coccidioides* clearance. Tissue-resident macrophages are one of the first responders in respiratory mediated fungal infections. They play a tolerogenic surveillance role, ensuring immune responses are effective but also not unnecessarily damaging to the delicate airway architecture. Complementing this tolerogenic role, alveolar macrophages, once activated, have a higher phagocytosis capability, higher OS production capacity, and are more adept at pathogen clearance [7]. Early phagocytic studies in rhesus macaque macrophages suggest this may be the case [27]. Alveolar macrophages played a role in *Cryptococcus neoformans* clearance, with some variability depending on laboratory strain used, whereas their interstitial macrophage counterparts are found to harbor the fungi intracellularly [28]. Phagocytosis increases when the alveolar macrophages are stimulated with LPS, suggesting pro-inflammatory signals are particularly beneficial for enhancing pathogen opsonization (Figure 1H). This was shown in previous studies where IFNγ enhanced phagocytosis rates in both peritoneal and alveolar mouse macrophages but only enhanced fungicidal killing within alveolar macrophages [29]. Our data and previous studies suggest that alveolar macrophages play an important role in *Coccidioides* infection control, and their function could be enhanced by strong pro-inflammatory signals. Further work must be done to help further characterize the specific mechanisms by which alveolar macrophages are more efficient killers than their non-tissue resident counterparts.

DCs play a critical role in activating and educating adaptive immune cells for a proper, effective immune response. Chronic disease often implies a breakdown either in adaptive immunity or earlier during innate immunity. With chronic coccidioidomycosis, one theory is that during disease clearance, DC polarization into DC2 would activate a non-productive adaptive immune response against *Coccidioides*, leading to chronic disease. This would indicate that DCs also are not functionally responding to *Coccidioides* despite upregulating the DC1 (CD11c+ CD8a+SIRPa−) phenotypic markers. Previous studies with *Coccidioides* showed that DCs upregulate CD86 and CD80 when co-cultured with *Coccidioides* and *Coccidioides* antigen lysate, however these studies did not look at the co-expression of MHC-II and CD86 to describe DC activation and maturation [30]. DC activation by the vaccine *Coccidioides* strain is protective and antigen primed DCs protected susceptible mouse strains from virulent challenge [10]. Our data and previous studies suggest a partial activation and response from DCs to *Coccidioides*, whether it be whole or antigen lysate components, but our data suggest a novel mechanism of virulence where *Coccidioides* may evade immunity by inhibiting innate immune cell activation and maturation. Given host susceptibility genetics, incomplete DC activation could impact adaptive immunity much more critically for susceptible versus resistant hosts, potentially explaining why despite the lack of co-expression of MHC-II and CD86 in our in vitro studies, the vaccine strain still provides protection for susceptible mouse strains [31]. Further, the in vitro assay creates a high-pathogen interaction that is unlikely to occur in vivo. This high antigen frequency may enhance the influence of *Coccidioides* virulence on innate immune cells, whereas in vivo multiple cell types (lung epithelium, innate lymphocytes) interact with *Coccidioides* likely at lower individual frequencies. The in vitro supernatant DC polarization experiments seem to support this speculation as decreases in activation marker expression frequency occurred only in the presence of *Coccidioides* and not in the indirect assays (Figure 3K–M). Our data suggest that the activation and polarization block is *Coccidioides* contact-dependent and physical interaction between DCs and arthroconidia are needed to inhibit MHC-II and CD86 co-expression. Further, arthroconidia appear to utilize a novel, contact-dependent mechanism to evade immune responses by preventing innate immune cell polarization and activation.

Despite polarizing to a Th1/Th17-favorable pro-inflammatory DC1 subset in response to *Coccidioides*, DC activation and maturation appears impaired. DC1s lacked the typical MHC-II and CD86 co-expression typical of an activated/matured antigen presenting cell. This suggests that while DCs respond to *Coccidioides* by differentiating, the process is incomplete potentially resulting in impaired functional capacity. One possible explanation for this is that despite receiving activating signals, DCs are prevented from upregulating costimulatory and maturation markers by arthroconidia-specific virulence factors that are expressed during the fungal switch to the spherule phase. We also observed DCs progressing into DC1, whereas their macrophage counterparts could not differentiate beyond M0. While both cell types poorly upregulate activation/maturation markers, DCs seemed to respond better, at least phenotypically, than macrophages. This could be partially explained by the fact that most DCs do not engage in phagocytosis. but rather utilize pinocytosis, while macrophages phagocytose whole pathogen [32]. These different methods of antigen uptake may partially explain why macrophages poorly progress beyond M0. Arthroconidia also remain alive inside mouse macrophages and may continue to influence macrophage activation and maturation [33].

DC1 and DC2 responses slightly increase in the lung by day 7 post-avirulent infection and the DCs are only mildly activated, somewhat replicating in vitro polarization in response to *Coccidioides*. Total DC frequency does not appear to increase within the lung or lung-draining lymph node by flow cytometry, imaging of lung tissue sections recapitulates these data. Immunohistochemistry scoring of lung tissue sections show no significant changes in intermediate MO, alveolar macrophage, or dendritic cell numbers but a statistically significant increase in neutrophils at day 1 post infection. Although these data may seem contradictory, it is known that less than 20% of cells are released from lung tissue by standard dissociation methods, providing a smaller picture of lung cellular changes when measured by flow cytometry. In immunohistochemistry, difficulties of locating *Coccidioides* in the lung due to the physical obstructions and tissue architecture complexities may also impact how truly representative the images are of an infection state. The modest increase in neutrophils based on image scoring aligns with previously observed increases in neutrophils within vaccine studies and pediatric patient data [34,35,36].

DC1 and DC2 responses increase in the lung in modest capacity and DCs overall upregulate MHC-II and CD86 co-expression by day 7 post-infection while macrophages fail to upregulate co-expression, somewhat replicating the in vitro data. Single-gating analysis shows CD86+ macrophage frequency in the draining lymph node decreases by day 7 post infection (Supplemental Figure S2B). This suggests that the *Coccidioides* inhibitory mechanisms acting against innate immune cells we observed in vitro are also modestly impactful in vivo. While the vaccine strains are protective in mouse models, translation of vaccines to humans has thus far been unsuccessful, although several groups are actively investigating translational strategies. Studies utilizing complement proteins or *Coccidioides* fragments as agonists enhance host immunity, suggesting that adjuvants may provide additional efficacy to live, attenuated vaccine strains [15,34,37]. This study uses an avirulent *Coccidioides* vaccine strain and may not reflect the innate immune activation found against virulent, wildtype strains. Much exciting and illuminating work has been done to demonstrate the protection and recruitment of adaptive immune responses to this avirulent strain. However, innate immune response as shaped by vaccination is equally important, as these cells coordinate and mold the quality and durability of the subsequent adaptive effector and memory subsets. This vaccine strain has been shown to be protective and induces a mixed Th1/Th2/Th17 memory response but the specific early innate immune mechanisms leading to and shaping these responses have not been well characterized [15,37]. Our study shows that macrophages and DCs in vitro appear to be blocked at various polarization phases and fail to upregulate activation/maturation markers CD86 and MHC-II, suggesting a novel virulence mechanism where *Coccidioides* arthroconidia inhibit DC activation and maturation. Our in vivo data showed modest mixed DC1/DC2 presence in the lungs post-infection and overall DC activation in lungs and dLN by day 7 post-infection, recapitulating observed protection capacity for the vaccine strain.

## 5. Conclusions

We sought to characterize the innate immune responses to NR-166 avirulent *Coccidioides posadasii* (*Δcts2/Δard1/Δcts3*) by characterizing how macrophages and dendritic cells response to *Coccidioides*. This strain is used widely in vaccine studies with protective responses in murine models, however, little is known about the innate immune responses to this strain. We found in our studies evidence for a novel *Coccidioides* virulence mechanism where macrophage and dendritic cell maturation/activation is impaired. Macrophage polarization halted at M0 stage with reduced activation/maturation. Dendritic cells polarized towards DC1 subtype but said DC1s had reduced activation/maturation even when co-cultured with LPS. These data suggest *Coccidioides* has a virulence mechanism inhibiting DC activation/maturation by impacting MHC-II and CD86 expression in a contact dependent manner between DCs and arthroconidia. Our in vivo study found a statistically significant overall DC activation in the lungs and draining lymph node by day 7 post-infection and a mixed DC1/DC2 recruitment in the lungs. Though there was an increase in macrophages in the lung at day 7 post-infection, there was no heightened activation. The varied immune cell behavior between in vivo and in vitro experiments likely stems from higher antigen exposure frequency in vitro compared to in vivo infection. This possibly explains why the in vivo data demonstrate mixed DC1/DC2 presence while the in vitro data show a DC1 bias. These findings while interesting, may or not hold up with virulent infection and warrants additional study. The avirulent (*Δcts2/Δard1/Δcts3*) strain was used to characterize activation behaviors and polarization biases in innate immune cells during vaccine-induced immune protection. Overall, the data demonstrate the vaccine strain’s protective capability and characterized a mixed DC1/DC2 response, a potential explanation for the mixed Th1/Th2/Th17 protection observed in previous vaccine studies. Future studies further characterizing the mechanism of this novel virulence blocking mechanism could yield therapeutic targets for enhancing innate immune cell responses to *Coccidioides* and open further avenues for innate immune cell-based vaccines.

## Data Availability

Data is contained within the article or supplementary material; additional information is available upon request.

## Supplementary Materials

The following are available online at https://www.mdpi.com/article/10.3390/jof7080630/s1, Figure S1: Short term *Coccidioides* causes subtle changes in peripheral blood. Figure S2: Single-gating of CD86 and MHC-II macrophages and DCs in the lung and dLN reveals subtle changes in population frequencies over short-term infection. Figure S3: Total DCs increase in lung dLN day 1 post-infection then drops at day 7 post-infection. Figures S4 and S5: Gating strategy for in vitro macrophage and DC polarization experiments, respectively.
