# Supplementary material for: Macrophage and Dendritic Cell Activation and Polarization in Response to Coccidioides posadasii Infection
Source: J Fungi (Basel). 2021 Aug 3;7(8):630. doi: 10.3390/jof7080630 (PMC8397226; doi:10.3390/jof7080630)
Supplement: Supplementary file 1 [file jof-07-00630-s001.zip › jof-1314500-supplementary.pdf]

Journal of *Fungi*

MDPI

Supplementary Material

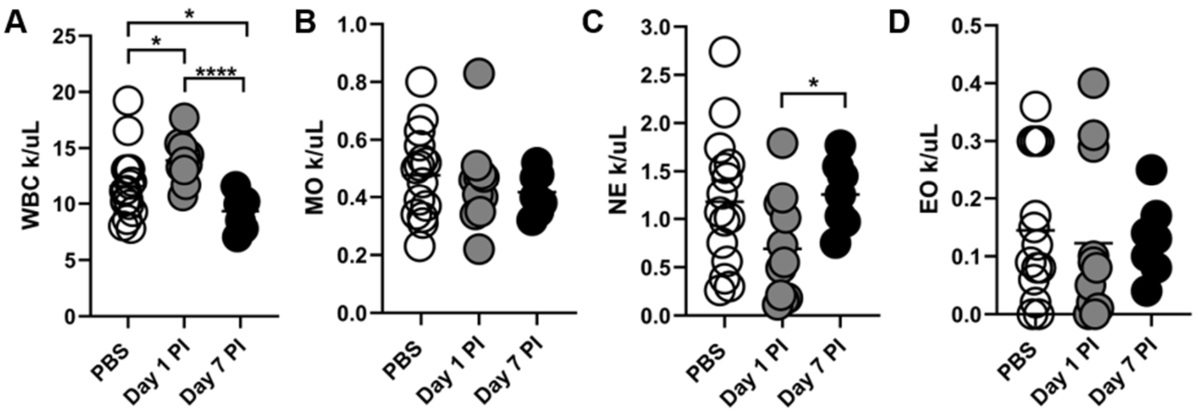

Figure S1. Short term *Coccidioides* infection causes subtle changes in peripheral blood. Peripheral blood analyzed using complete blood count. (**A**) WBC= white blood cell; (**B**) MO= monocyte; (**C**) NE= neutrophil; (**D**) EO= eosinophil. For all plots displayed: line indicates mean and each dot is one experimental replicate. Data was analyzed using a paired Student's T-test and outliers excluded using Grubbs Outlier exclusion analysis. *p<0.05, ****p<0.0005.

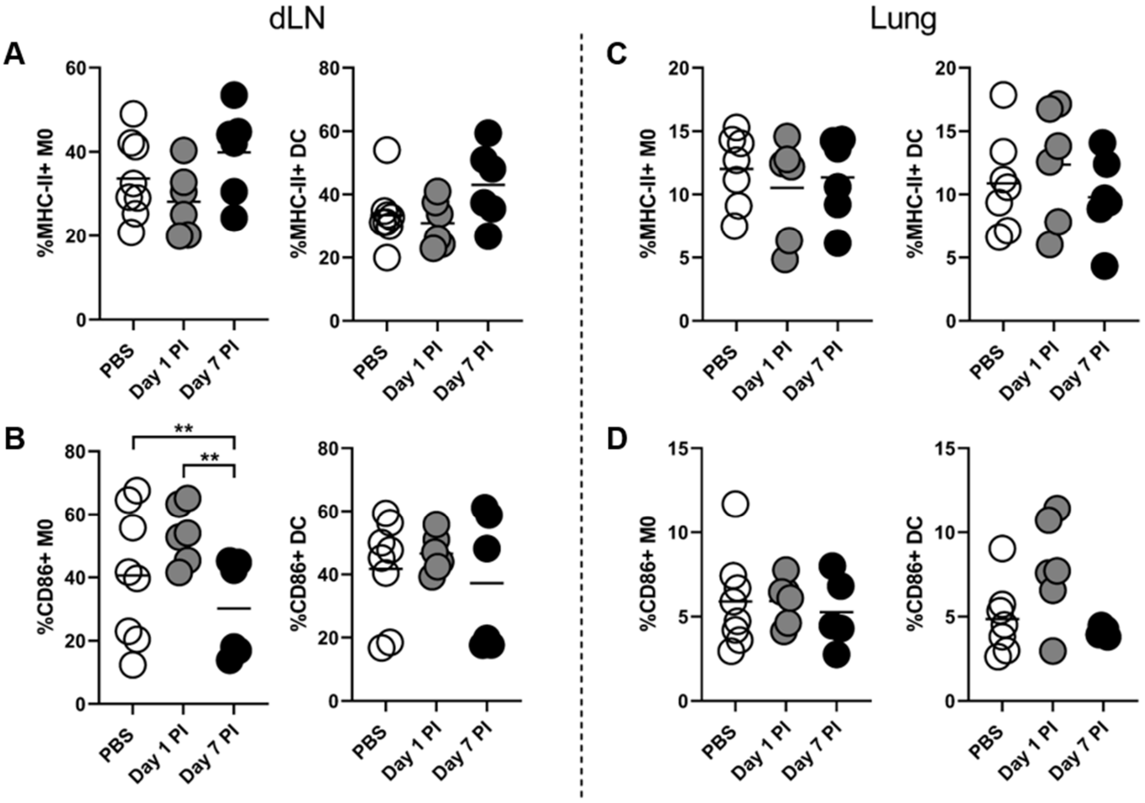

Figure S2. CD86+ macrophage frequency in the dLN decreases by day 7 post-infection. PBS was used as mock intranasal infection. 10^5 arthroconidia in 30uL PBS was used for Day 1 and Day 7 post-infection. (**A**) MHC-II+ M0 and DC frequencies in dLN. (**B**) CD86+ M0 and DC frequency in dLN. (**C**) MHC-II+ M0 and DC frequencies in lung. (**D**) CD86+ M0 and DC frequencies in the lung. For all plots displayed: line indicates mean and each dot is one experimental replicate. Data was analyzed using a paired Student's T-test and outliers excluded using Grubbs Outlier exclusion analysis.**p<0.005.

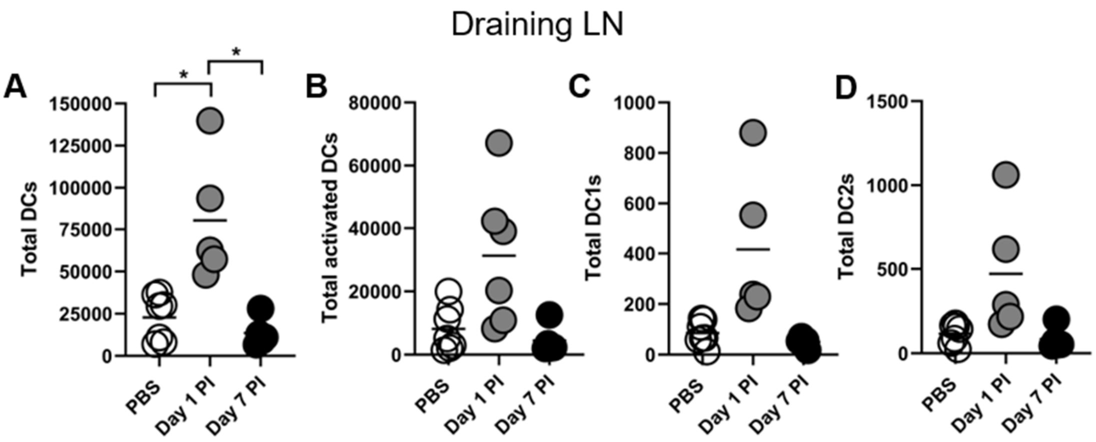

Figure S3. Total DCs increase in lung dLN day 1 post-infection then drops at day 7 post-infection. PBS was used as mock intranasal infection. 10^5 arthroconidia in 30uL PBS was used for Day 1 and Day 7 post-infection. (**A**) Total DCs in whole dLN (**B**) total activated DCs. (**C**) total DC1s (**D**) total DC2s.For all plots displayed: line indicates mean and each dot is one experimental replicate. Data was analyzed using a paired Student's T-test and outliers excluded using Grubbs Outlier exclusion analysis. **p*<0.05.

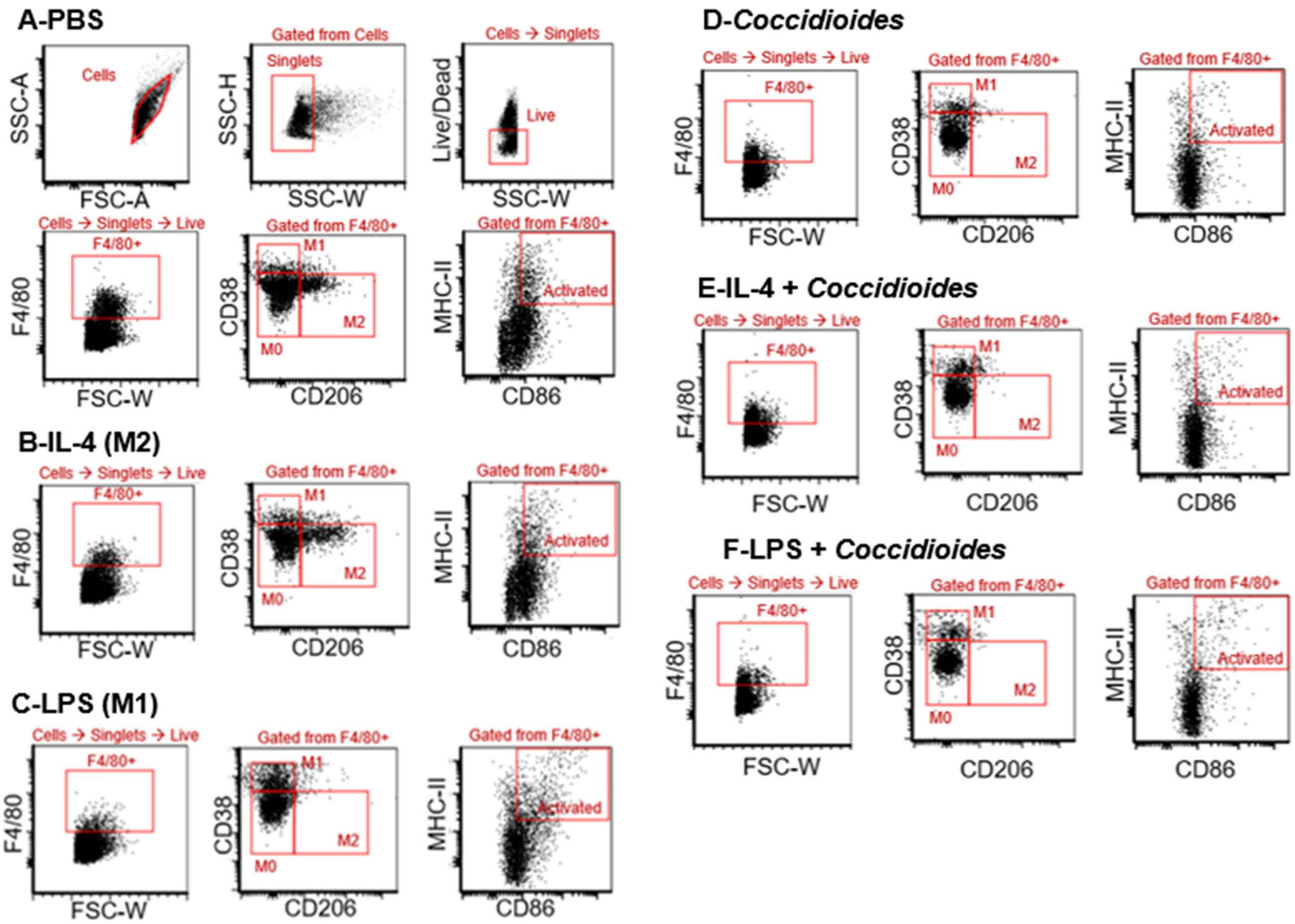

Figure S4. Gating strategy for in vitro macrophage polarization experiments. Debris gate → singlets → live → F4/80+ → M0-M1-M2 → Activated. Representative gating plots for each of the following conditions as labeled (**A**) PBS aka no stimulation (**B**) added 20 ng/mL IL-4 aka M2 condition, (**C**) added 100 ng/mL LPS aka M1 condition, (**D**) *Coccidioides*, (**E**) IL-4

and *Coccidioides*, and (**F**) LPS and *Coccidioides.* SSCA- vs. FSC-A used was recorded in log to capture all large cells instead of the typical linear data capture. M0/M1/M2 cells are gated from the F4/80+ gate and are defined as such: M0 F4/80+ CD38- CD206-, M1 F4/80+ CD38+ CD206-, and M2 F4/80+ CD38- CD206+. The activated gate is then gated off the respective F4/80+ (macrophages), M0, M1, and M2 populations from the previous gate to give population frequencies used in Figure 2.

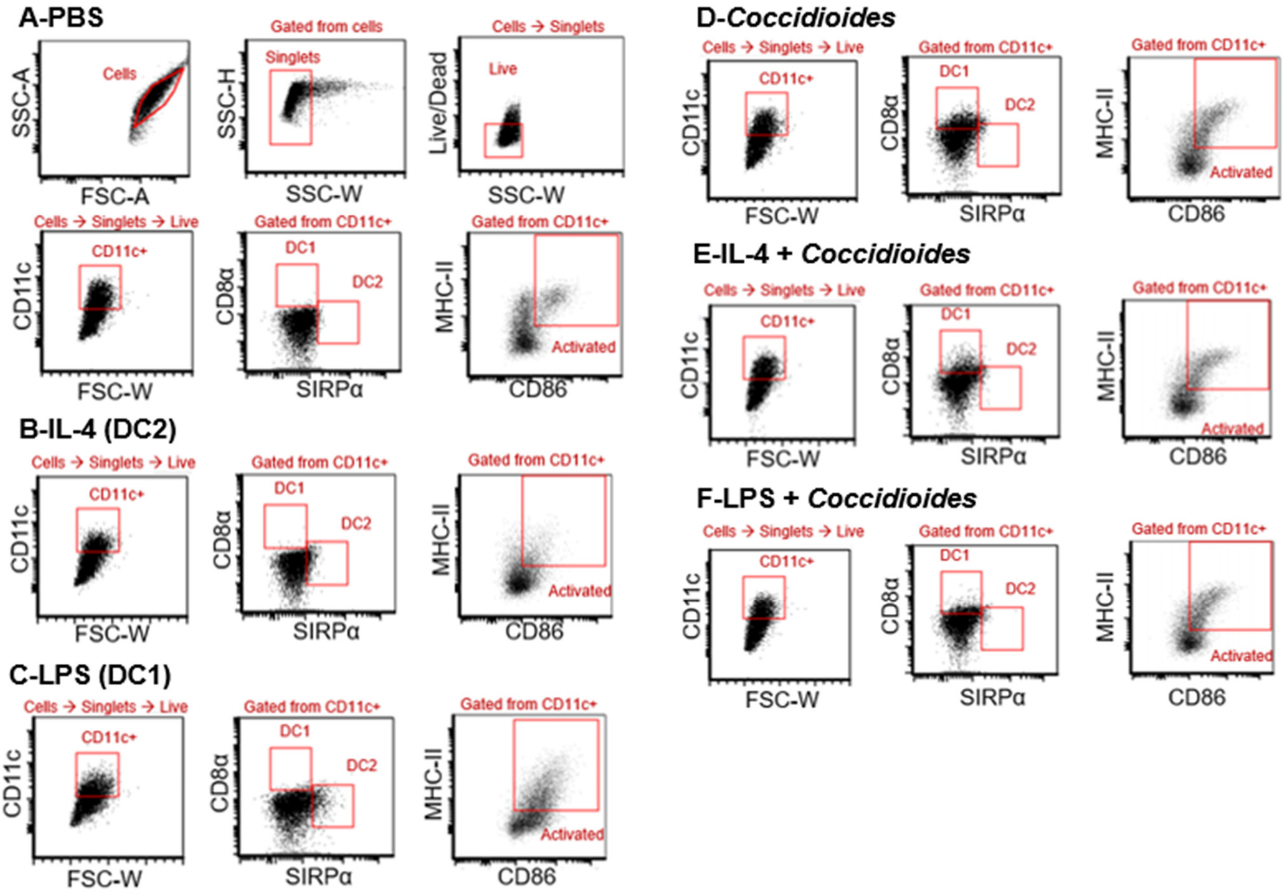

Figure S5. Gating strategy for in vitro dendritic cell polarization experiments. Debris gate → singlets → live → CD11c+ → DC1-DC2 → Activated. Representative gating plots for each of the following conditions as labeled (**A**) PBS aka no stimulation (**B**) added 20 ng/mL IL-4 aka DC2 condition, (**C**) added 100 ng/mL LPS aka DC1 condition, (**D**) *Coccidioides*, (**E**) IL-4 and *Coccidioides*, and (**F**) LPS and *Coccidioides.* SSCA- vs. FSC-A used was recorded in log to capture all large cells instead of the typical linear data capture. DC1/DC2 cells are gated from the CD11c+ gate and are defined as such: DCs are total CD11c+ populations, DC1 CD11c+ CD8a+ SIRPa-, DC2 CD11c+ CD8a- SIRPa+. The activated gate is then gated off the respective CD11c+ (dendritic cells), DC1, and DC2 populations from the previous gate to give population frequencies used in Figure 3.
